# Comparative risk of psychiatric comorbidities associated with codeine and tramadol in patients with hip osteoarthritis: a nationwide population-based cohort study

**DOI:** 10.7189/jogh.16.04121

**Published:** 2026-04-24

**Authors:** Yujin Kim, Eunjung Choo, Sooyoung Shin, Yeo Jin Choi

**Affiliations:** 1 Department of Regulatory Science, Graduate School, Kyung Hee University, Seoul, Republic of Korea; 2 Institute of Regulatory Innovation through Science, Kyung Hee University, Seoul, Republic of Korea; 3 Department of Pharmacy, College of Pharmacy and Institute of Integrated Pharmaceutical Sciences, Kyung Hee University, Seoul, Republic of Korea; 4 Department of Pharmacy, College of Pharmacy, Ajou University, Suwon, Republic of Korea; 5 Department of Biohealth Regulatory Science, Graduate School, Ajou University, Suwon, Republic of Korea

## Abstract

**Background:**

Weak opioids are often prescribed for osteoarthritis (OA), yet their comparative psychiatric risks are not well established. We aimed to comprehensively compare the composite psychiatric risks associated with codeine and tramadol in patients diagnosed with hip OA.

**Methods:**

We conducted a nationwide, population-based retrospective cohort study, using Korean Health Insurance Review and Assessment Service database on patients diagnosed with hip OA between 2014 and 2017. We included patients who received either opioid, with a total of 22 651 patients (of whom 4533 codeine and 18 118 tramadol users) after 1:4 propensity score matching (PSM). We applied Cox proportional hazards models to estimate adjusted hazard ratios (aHR) and 95% confidence intervals (CIs) for incident psychiatric outcomes.

**Results:**

Codeine use was associated with a significantly lower hazard of composite psychiatric disorders (aHR = 0.86; 95% CI = 0.78–0.96), particularly anxiety (aHR = 0.81; 95% CI = 0.69–0.95), and showed a borderline reduction in sleep disorders (aHR = 0.81; 95% CI = 0.65–1.00, *P* = 0.048) after adjustment for age, sex, comorbidities, and concomitant medications. Subgroup analyses revealed consistently lower psychiatric risk among patients with a high comorbidity burden (Charlson’s comorbidity index ≥3), cardiovascular disease, or those without concomitant psychotropic medications. Sensitivity analyses using inverse probability treatment weighting and 1:1 PSM demonstrated broadly similar patterns, although statistical significance varied across models. No clear duration-response relationship was observed.

**Conclusions:**

Codeine was associated with lower hazards of anxiety and sleep disorders in several analyses. These findings suggest that strengthening opioid stewardship through structured psychiatric risk assessment and individualised prescribing may enhance patient safety. Further controlled studies incorporating detailed clinical data are warranted to validate these associations and to better define their implications for long-term opioid management and policy development.

Osteoarthritis (OA) is one of the most prevalent chronic degenerative musculoskeletal diseases and a leading cause of disability and pain, affecting approximately 595 million people worldwide in 2020 [1]. Hip OA often results in more severe pain, functional impairment, and reduced quality of life [2,3]. These patients may also experience concomitant knee pain without radiographic evidence of knee involvement, complicating pain assessment and management [2,3]. Current clinical guidelines recommend nonsteroidal anti-inflammatory drugs or acetaminophen as first-line analgesics, reserving opioids for short-term management of severe or refractory pain [4]. Nonetheless, weak opioids, such as codeine and tramadol, are often prescribed long-term, raising concerns regarding central nervous system (CNS) effects, risk of misuse, and other complications [4,5].

Previous studies have linked opioid use, including non-medical prescription opioids, to an increased risk of mood and anxiety disorders [6]. Long-term opioid use (≥180 days) has been associated with a 51% higher depression risk in the USA veterans, and duration of use appears to be a stronger predictor of depression than dosage [7,8]. Furthermore, the Opioid Risk Tool, designed to assess the risk of opioid misuse in chronic pain patients, identifies psychiatric disorders such as attention-deficit disorder, bipolar disorder, depression, and schizophrenia as key predictors of opioid misuse [9]. These findings imply a potential mutual link between psychiatric disorders and opioid use, emphasising the need for real-world evidence to guide safer opioid prescribing strategies, particularly in individuals who are vulnerable to severe, persistent chronic pain.

Tramadol, despite its μ-opioid receptor agonist activity, has become one of the most widely used analgesics in clinical practice [10,11]. Tramadol prescribing has grown significantly not only in Korea, where its use is especially widespread among older adults, but also in the USA, where utilisation among patients with OA continues to rise [10,11]. However, its dual mechanism, μ-opioid receptor agonism and inhibition of serotonin and norepinephrine reuptake, may predispose users to neuropsychiatric effects [10,12]. Although both tramadol and codeine are classified as weak opioids and used for persistent musculoskeletal pain, their pharmacological profiles differ markedly, raising the possibility of differential neuropsychiatric risks. Yet, real-world evidence on comparative neuropsychiatric safety remains limited. Therefore, we aimed to fill this critical knowledge gap by comparing the risk of psychiatric disorders associated with codeine and tramadol use in a nationwide population-based cohort of patients with hip OA, thereby providing essential evidence to refine opioid selection and clinical decision-making in chronic pain management.

## METHODS

### Data source and study population

We conducted this nationwide population-based retrospective cohort study using the Health Insurance Review and Assessment Service National Claims Data (M20230724004), following the STROBE guidelines [13]. The database contains administrative data from the National Health Insurance related to healthcare services, covering demographics, healthcare institution type, outpatient and inpatient encounters, admission details, diagnoses, and prescription records [14]. Diagnoses are coded using the Korean Standard Classification of Diseases 8th revision, adapted from the International Classification of Diseases, tenth revision (ICD-10). As we relied on a nationwide administrative claims database with mandatory recording of diagnoses and prescriptions, missing data for exposures, outcomes, and covariates were minimal. Therefore, we did not undertake any imputation procedures, and analyses were based on complete-case data.

**Figure 1:**
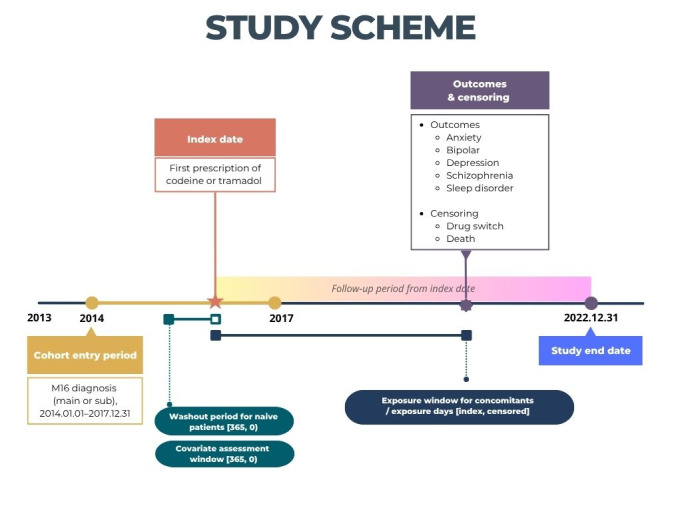
Study scheme. Washout period indicates a pre-exposure window used to ensure inclusion of naïve patients with no prior opioid use; the covariate assessment window indicates the period before the index date used to evaluate baseline characteristics (*e.g.* comorbidities, demographics); and the exposure window indicates the observation period beginning at the start of tramadol or codeine use.

Patients diagnosed with hip OA (ICD-10 code M16) between January 2014 and December 2017 were eligible for inclusion if they were prescribed either codeine or tramadol (**Figure 1**). We defined the index date as the first prescription date of codeine or tramadol following a hip OA diagnosis. We selected tramadol as the index exposure because it is the most prescribed weak opioid in real-world musculoskeletal pain management and possesses a distinct dual mechanism, which may confer differential neuropsychiatric effects. Codeine served as the active comparator because it represents traditional weak opioid use for similar indications. We excluded individuals who had any psychiatric disorder diagnosis before the index date; those who had a medication history of buprenorphine or naloxone injection within 12 months prior to the index date; those with a history of opioid related disorder (F11); those younger than 20 and older than 85 years; those with a cancer diagnosis (C00–D48); who had a low dihydrocodeine dose (<50 mg), which is typically used as antitussive; and those with any prescription of tramadol or codeine within 12 months prior to the index date. As we utilised a nationwide claims database and included all eligible patients during the study period, we did not perform a formal *a priori* sample size or power calculation.

### Primary outcomes

We defined psychiatric outcomes using ICD-10 diagnostic codes aligned with the corresponding Diagnostic and Statistical Manual of Mental Disorders, fifth edition disorder categories. Our primary outcome was the incidence of overall composite psychiatric comorbidities, including depression (F32–33), anxiety disorder (F40, F41, F42), bipolar disorder (F30–31), schizophrenia (F20–25), and sleep disorder (F51).

### Secondary outcomes

We stratified two secondary composite outcomes: composite 1 (including anxiety disorders, depression, and sleep disorders), representing psychiatric conditions with relatively shorter clinical latency and composite 2 (bipolar disorder and schizophrenia), representing severe psychiatric disorders with longer latency and lower incidence.

### Follow-up and covariates

We followed patients from the index date until the earliest of the following: outcome occurrence, death, switch to another opioid, or end of follow-up (31 December 2022), whichever occurred first. The maximum follow-up period was nine years. We assessed baseline characteristics, including demographics, sex, age, Charlson Comorbidity Index (CCI), comorbidities, and comedications, during the 12 months preceding the index date. We defined comedications as drugs prescribed for a cumulative duration of at least 30 days within this 12-month period.

### Statistical analysis

To compare the risk of psychiatric disorder between codeine and tramadol users, we applied propensity score-matching (PSM) using 1:4 nearest-neighbour matching without replacement and a calliper width of 0.1. We assessed covariate balance using standardised mean differences (SMDs), with SMD<0.1 considered indicative of adequate balance. We estimated cumulative incidence rates (IRs) using Kaplan-Meier curves and compared them via the log-rank test. As the primary objective was to compare time to incident psychiatric outcomes while accounting for variable follow-up duration and censoring due to death, opioid switching, or end of follow-up, we used Cox proportional hazards models to estimate hazard ratios (HR) with 95% confidence intervals (CIs). We adjusted the primary Cox model for a core set of prespecified covariates, including age, sex, CCI, baseline comorbidities, and key concomitant medications. To further address potential residual confounding, we performed a fully adjusted Cox proportional hazards model incorporating all prespecified baseline covariates, including detailed comorbidities and concomitant medications assessed during the 12 months prior to cohort entry, as a sensitivity analysis. We determined the covariates included in both models *a priori* based on prior clinical and epidemiological knowledge and used no data-driven variable selection procedures. We examined the proportional hazards assumption using Schoenfeld residuals and observed no substantial violations. We also visually inspected the Kaplan-Meier curves, which further supported the appropriateness of the proportional hazard methodology and the consistency of model-independent absolute risk estimates. We performed subgroup analyses in the matched cohort by sex, age group (20–39, 40–64, and ≥65 years), CCI, insurance type, comorbidities, and comedications. Additionally, we conducted sensitivity analyses using alternative adjustment strategies, including 1:1 PSM and inverse probability of treatment weighting (IPTW). Also, to evaluate the potential influence of exposure duration, we conducted supplementary sensitivity analyses within the originally constructed 1:4 PSM cohort by restricting the analysis to patients with cumulative opioid use of ≥7, ≥14, ≥30, and ≥60 days following the index prescription.

We used SAS, version 7.14 (SAS Institute, Inc., Cary, North Carolina, USA) and *R*, version 3.5.1 (R Core Team, Vienna, Austria) for all analyses.

## RESULTS

### Patient characteristics

**Figure 2:**
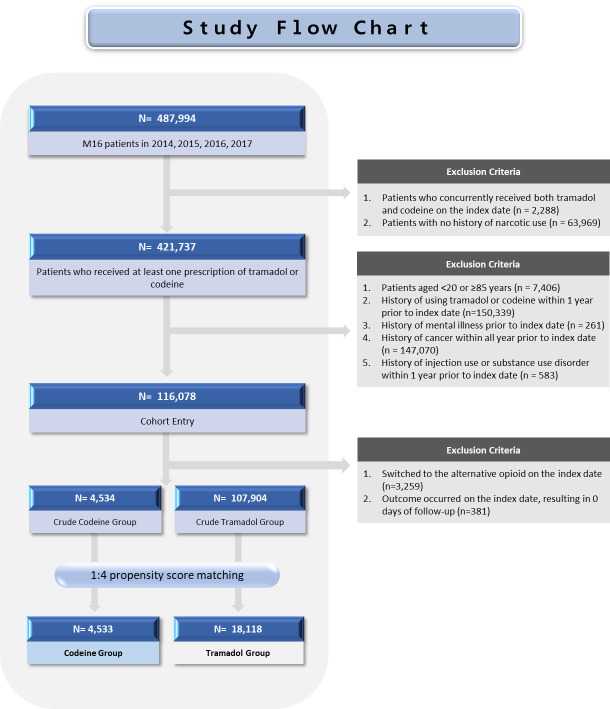
Flowchart of study population selection.

Among 487 994 patients diagnosed with hip OA between 2014 and 2017, 421 737 patients received at least one prescription for either codeine or tramadol (**Figure 2**). A total of 116 078 patients met the eligibility criteria and were included in the final cohort. After 1:4 PSM, we retained 22 651 patients, of whom 4533 were codeine and 18 118 tramadol users (**Table 1**). The age distribution was comparable between groups, with approximately 44.7% of patients aged ≥65 years. The mean CCI scores were 2.03 (standard deviation = 1.83) in the codeine and 2.01 (standard deviation = 1.89) in the tramadol group. Overall, baseline demographic characteristics, comorbidities, and comedications were generally well balanced between the two groups after matching, with SMD<0.1 for all covariates except musculoskeletal disorders other than osteoporosis and rheumatoid arthritis.

**Table 1:** Baseline characteristics of the PS-matched cohort*

	Before PSM			After PSM		
	**Codeine (n = 4534)**	**Tramadol (n = 107 904)**	**SMD**	**Codeine (n = 4533)**	**Tramadol (n = 18 118)**	**SMD**
**Age**			0.084			0.026
20–24	60 (1.3)	1436 (1.3)		60 (1.3)	249 (1.4)	
25–29	140 (3.1)	2496 (2.3)		140 (3.1)	529 (2.9)	
30–34	130 (2.9)	3031 (2.8)		130 (2.9)	523 (2.9)	
35–39	180 (4.0)	3895 (3.6)		180 (4.0)	671 (3.7)	
40–44	217 (4.8)	5131 (4.8)		217 (4.8)	837 (4.6)	
45–49	315 (6.9)	7192 (6.7)		315 (6.9)	1292 (7.1)	
50–54	395 (8.7)	10 346 (9.6)		395 (8.7)	1567 (8.6)	
55–59	508 (11.2)	13 142 (12.2)		508 (11.2)	2087 (11.5)	
60–64	569 (12.5)	13 505 (12.5)		569 (12.6)	2270 (12.5)	
65–69	508 (11.2)	12 922 (12.0)		508 (11.2)	2066 (11.4)	
70–74	567 (12.5)	12 933 (12.0)		567 (12.5)	2298 (12.7)	
75–79	513 (11.3)	12 872 (11.9)		513 (11.3)	2060 (11.4)	
80–84	432 (9.5)	9003 (8.3)		431 (9.5)	1669 (9.2)	
**Sex**			0.037			0.004
Male	1918 (42.3)	47 625 (44.1)		1918 (42.3)	7634 (42.1)	
Female	2616 (57.7)	60 279 (55.9)		2615 (57.7)	10 484 (57.9)	
**CCI, x̄ (SD)**	2.03 (1.83)	1.66 (1.72)	0.209	2.03 (1.83)	2.01 (1.89)	0.011
**CCI**			0.247			0.091
0	912 (20.1)	32 385 (30.0)		912 (20.1)	4164 (23.0)	
1	1220 (26.9)	29 081 (27.0)		1220 (26.9)	4686 (25.9)	
2	984 (21.7)	19 726 (18.3)		984 (21.7)	3570 (19.7)	
3	620 (13.7)	11 729 (10.9)		620 (13.7)	2268 (12.5)	
≥4	798 (17.6)	14 983 (13.9)		797 (17.6)	3430 (18.9)	
**Index year**			0.209			0.025
2014	340 (7.5)	10 360 (9.6)		340 (7.5)	1368 (7.6)	
2015	618 (13.6)	16 363 (15.2)		618 (13.6)	2448 (13.5)	
2016	856 (18.9)	19 270 (17.9)		856 (18.9)	3431 (18.9)	
2017	932 (20.6)	20 542 (19.0)		932 (20.6)	3806 (21.0)	
2018	804 (17.7)	14 308 (13.3)		803 (17.7)	3199 (17.7)	
2019	492 (10.9)	10 099 (9.4)		492 (10.9)	1997 (11.0)	
2020	208 (4.6)	6543 (6.1)		208 (4.6)	832 (4.6)	
2021	129 (2.8)	5381 (5.0)		129 (2.8)	463 (2.6)	
2022	155 (3.4)	5038 (4.7)		155 (3.4)	574 (3.2)	
**Insurance type**			0.023			0.004
Health insurance	4246 (93.6)	101 107 (93.7)		4245 (93.6)	16 953 (93.6)	
Medical aid	276 (6.1)	6623 (6.1)		276 (6.1)	1114 (6.1)	
Veterans' medical benefits	12 (0.3)	174 (0.2)		12 (0.3)	51 (0.3)	
**Comorbidities**						
Hypertension	2084 (46.0)	47 127 (43.7)	0.046	2083 (46.0)	8295 (45.8)	0.003
Dyslipidaemia	2155 (47.5)	47 247 (43.8)	0.075	2154 (47.5)	8660 (47.8)	0.006
Arrhythmia	267 (5.9)	4085 (3.8)	0.098	266 (5.9)	816 (4.5)	0.062
Heart Failure	283 (6.2)	3932 (3.6)	0.12	282 (6.2)	924 (5.1)	0.049
Myocardial infarction	645 (14.2)	11 316 (10.5)	0.114	644 (14.2)	2290 (12.6)	0.046
CVD	2825 (62.3)	63 869 (59.2)	0.064	2824 (62.3)	11 335 (62.6)	0.005
DM	1248 (27.5)	27 325 (25.3)	0.05	1247 (27.5)	4998 (27.6)	0.002
Stroke	334 (7.4)	6932 (6.4)	0.037	333 (7.3)	1236 (6.8)	0.02
CKD	99 (2.2)	1604 (1.5)	0.052	99 (2.2)	380 (2.1)	0.006
COPD	261 (5.8)	2795 (2.6)	0.159	260 (5.7)	882 (4.9)	0.039
Asthma	1122 (24.7)	14 002 (13.0)	0.304	1122 (24.8)	4453 (24.6)	0.004
Dyspnoea	374 (8.2)	4207 (3.9)	0.183	374 (8.3)	1323 (7.3)	0.035
Respiratory disease	1418 (31.3)	17 984 (16.7)	0.347	1417 (31.3)	5661 (31.2)	<0.001
Pulmonary oedema	17 (0.4)	194 (0.2)	0.037	17 (0.4)	38 (0.2)	0.031
Other musculoskeletal disorders	2589 (57.1)	65 854 (61.0)	0.08	2589 (57.1)	11 505 (63.5)	0.131
Rheumatic disease	241 (5.3)	6061 (5.6)	0.013	241 (5.3)	983 (5.4)	0.005
Osteoporosis	1031 (22.7)	21 721 (20.1)	0.064	1030 (22.7)	4165 (23.0)	0.006
Parkinson disease	82 (1.8)	1685 (1.6)	0.019	82 (1.8)	348 (1.9)	0.008
Alzheimer disease	34 (0.7)	611 (0.6)	0.023	34 (0.8)	141 (0.8)	0.003
Epilepsy	112 (2.5)	1975 (1.8)	0.044	112 (2.5)	401 (2.2)	0.017
Neurological disorder	206 (4.5)	3831 (3.6)	0.05	206 (4.5)	796 (4.4)	0.007
Peripheral vascular disease	571 (12.6)	15 970 (14.8)	0.064	571 (12.6)	2280 (12.6)	<0.001
Sleep disorder	552 (12.2)	10 231 (9.5)	0.087	551 (12.2)	2062 (11.4)	0.024
Hypothyroidism	201 (4.4)	4415 (4.1)	0.017	201 (4.4)	834 (4.6)	0.008
Hyperthyroidism	78 (1.7)	1539 (1.4)	0.024	78 (1.7)	312 (1.7)	<0.001
Thyroid disorder	264 (5.8)	5699 (5.3)	0.024	264 (5.8)	1091 (6.0)	0.008
Chronic liver disease	156 (3.4)	3176 (2.9)	0.028	156 (3.4)	694 (3.8)	0.021
Peptic ulcer disease	1263 (27.9)	28 179 (26.1)	0.039	1263 (27.9)	5890 (32.5)	0.101
GERD	2521 (55.6)	48 963 (45.4)	0.206	2520 (55.6)	9519 (52.5)	0.061
IBD	10 (0.2)	164 (0.2)	0.016	10 (0.2)	33 (0.2)	0.009
Gastrointestinal disorder	2974 (65.6)	61 085 (56.6)	0.185	2973 (65.6)	11 972 (66.1)	0.01
Medication abuse	6 (0.1)	96 (0.1)	0.013	6 (0.1)	19 (0.1)	0.008
Fracture	604 (13.3)	12 113 (11.2)	0.064	603 (13.3)	2448 (13.5)	0.006
**Concomitant medications**						
Benzodiazepines	633 (14.0)	14 127 (13.1)	0.025	633 (14.0)	2555 (14.1)	0.004
Anticonvulsants	66 (1.5)	1423 (1.3)	0.012	66 (1.5)	269 (1.5)	0.002
Antidepressants	336 (7.4)	6521 (6.0)	0.055	335 (7.4)	1351 (7.5)	0.003
Antipsychotics	115 (2.5)	1935 (1.8)	0.051	114 (2.5)	446 (2.5)	0.003
Hypnotics	159 (3.5)	3203 (3.0)	0.03	159 (3.5)	573 (3.2)	0.019
NSAIDs	1863 (41.1)	43 433 (40.3)	0.017	1863 (41.1)	7550 (41.7)	0.012
Acetaminophen	430 (9.5)	7857 (7.3)	0.08	430 (9.5)	1614 (8.9)	0.02
Corticosteroids	312 (6.9)	5807 (5.4)	0.063	312 (6.9)	1273 (7.0)	0.006

CCI – Charlson Comorbidity Index, CKD – chronic kidney disease, COPD – chronic obstructive pulmonary disease, CVD – cardiovascular disease, DM – diabetes mellitus, GERD – gastroesophageal reflux disease, IBD – inflammatory bowel disease, NSAID – nonsteroidal anti-inflammatory drug, PSM – propensity score matching, PVD – peripheral vascular disease, SD – standard deviation, SMD – standardised mean differences, x̄ – mean

*Variables are presented as numbers (percentages) unless specified otherwise.

### Risk of psychiatric diseases

**Figure 3:**
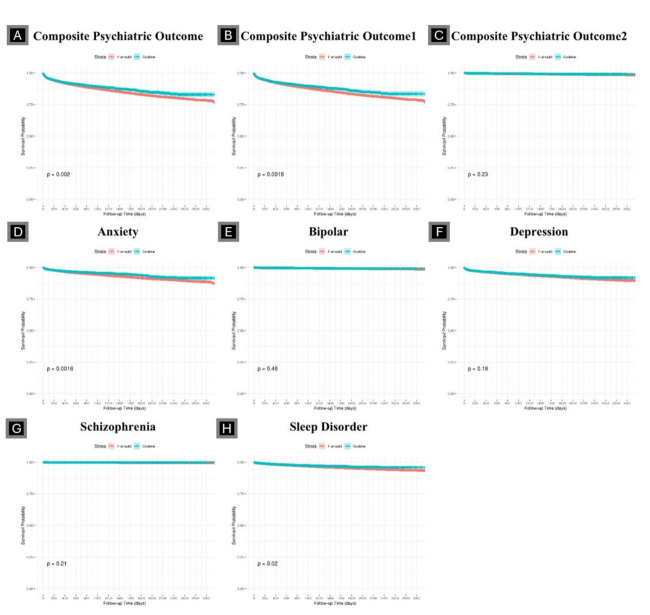
Kaplan-Meier curves of psychiatric outcomes. **Panel A.** Composite psychiatric outcome. **Panel B.** Composite psychiatric outcome 1. **Panel C.** Composite psychiatric outcome 2. **Panel D.** Anxiety. **Panel E.** Bipolar. **Panel F.** Depression. **Panel G.** Schizophrenia. **Panel H.** Sleep disorder.

Codeine use was associated with a lower hazard of anxiety and sleep disorders compared with tramadol use, whereas no statistically significant differences were observed for depression, bipolar disorder, or schizophrenia (**Figure 3**, Panels A–H). The IR per 1000 person-years (PY) of composite psychiatric disorders was 38.9 among tramadol users and 39.9 among codeine users (**Table 2**). In time-to-event analyses accounting for differential follow-up and censoring, codeine use was associated with a lower adjusted hazard compared with tramadol (adjusted HR (aHR) = 0.86; 95% CI = 0.78–0.96). The IR for anxiety was 15.7 per 1000 PY in codeine users and 17.6 in tramadol users (aHR = 0.81; 95% CI = 0.69–0.95). This reflected an absolute difference of approximately 1.9 fewer anxiety-related events per 1000 PY among codeine users (IR difference = −1.9 per 1000 PY). The IR of bipolar disorder, depression, and schizophrenia were comparable between groups, with no statistically significant differences in aHR. The IR for sleep disorder was 8.65 in the codeine and 9.65 in the tramadol group (aHR = 0.81; 95% CI = 0.65–1.00, *P* = 0.048). In absolute terms, this represented approximately 1.0 fewer sleep disorder events per 1000 PY among codeine users compared with tramadol users (IR difference = −1.0 per 1000 PY).

**Table 2:** Risk of psychiatric disorders and all-cause mortality in codeine *vs.* tramadol users

	Codeine			Tramadol								
	**n**	**Total PY**	**IR/1000PY**	**n**	**Total PY**	**IR/1000PY**	**HR (95% CI)**	***P*-value**	aHR (95%CI)*	***P*-value**	**aHR (95% CI)**†	***P*-value**
**Composite psychiatric outcome all**	428	10 720.3	39.9	2802	72 114.8	38.9	0.85 (0.77–0.94)	0.002	0.86 (0.77–0.95)	0.003	0.86 (0.78–0.96)	0.005
**Composite psychiatric outcome 1**	412	10 747.5	38.3	2728	72 400.5	37.7	0.85 (0.76-0.94)	0.002	0.86 (0.78–0.96)	0.006	0.87 (0.78–0.96)	0.008
**Anxiety**	177	11 265.1	15.7	1358	77 118.6	17.6	0.78 (0.66–0.91)	0.002	0.80 (0.68–0.94)	0.006	0.81 (0.69–0.95)	0.001
**Depression**	202	11 236.6	18.0	1277	77 295.9	16.5	0.89 (0.77–1.04)	0.157	0.93 (0.80–1.08)	0.330	0.92 (0.79–1.07)	0.294
**Sleep disorder**	99	11 450.9	8.65	763	79 037.1	9.65	0.78 (0.63–0.96)	0.020	0.80 (0.65–0.98)	0.036	0.81 (0.65–1.00)	0.048
**Composite psychiatric outcome 2**	23	11 616.8	1.98	172	80 847.7	2.18	0.77 (0.50–1.19)	0.232	0.76 (0.49–1.18)	0.217	0.72 (0.46–1.12)	0.145
**Bipolar disorder**	19	11 626.1	1.63	135	80 985.2	1.67	0.83 (0.51–1.35)	0.461	0.83 (0.51–1.36)	0.465	0.79 (0.49–1.30)	0.358
**Schizophrenia**	5	11 650.3	0.43	47	81 248.5	0.58	0.56 (0.22–1.41)	0.218	0.58 (0.23–1.48)	0.254	0.54 (0.21–1.39)	0.201

aHR – adjusted hazard ratio, CI – confidence interval, HR – hazard ratio, IR – incidence rate, PY – person-years

*Adjusted for age, sex, Charlson Comorbidity Index, index year, insurance type, history of sleep disorder, and concomitant medication use (benzodiazepines, anticonvulsants, antidepressants, antipsychotics, hypnotics, AAP, nonsteroidal anti-inflammatory drugs, and corticosteroids).

†Adjusted for age, sex, index year, insurance type, Charlson Comorbidity Index, comorbidities (hypertension, dyslipidaemia, arrhythmia, heart failure, myocardial infarction, cardiovascular disease, diabetes mellitus, stroke, chronic kidney disease, chronic obstructive pulmonary disease, asthma, dyspnoea, other respiratory diseases, pulmonary oedema, musculoskeletal diseases, rheumatic diseases, osteoporosis, Parkinson disease, Alzheimer disease, epilepsy, neurological disorders, peripheral vascular disease, hyperthyroidism, hypothyroidism, liver disease, peptic ulcer disease, gastroesophageal reflux disease, inflammatory bowel disease, and gastrointestinal diseases), history of medication abuse and fracture, history of sleep disorder, and concomitant medication use (benzodiazepines, anticonvulsants, antidepressants, antipsychotics, hypnotics, psychotropic drugs, non-opioid analgesics, nonsteroidal anti-inflammatory drugs, AAP, and corticosteroids).

### Subgroup analyses

Codeine use was associated with a lower hazard of composite psychiatric outcome in patients with higher comorbidity burden (CCI≥3) (HR = 0.81; 95% CI = 0.68-0.96) and those with cardiovascular disease (CVD) (HR = 0.78; 95% CI = 0.68–0.88) (**Table 3**). Lower hazard estimates were also observed among patients without concomitant use of benzodiazepine (HR = 0.84; 95% CI = 0.74–0.96), antiepileptics (HR = 0.85; 95% CI = 0.77–0.94), antidepressants (HR = 0.82; 95% CI = 0.72–0.92), antipsychotics (HR = 0.85; 95% CI = 0.77–0.95), hypnotics (HR = 0.84; 95% CI = 0.76–0.94), and corticosteroids (HR = 0.86; 95% CI = 0.78–0.96). For anxiety, a similar trend was observed across most subgroups. Statistically significant associations for anxiety (HR = 0.78; 95% CI = 0.66-0.91) and sleep disorders (HR = 0.77; 95% CI = 0.62–0.95) were observed among patients without chronic kidney disease. There were no statistically significant differences between groups for bipolar disease or depression across subgroups.

**Table 3:** IR and HR for the composite psychiatric outcome by subgroup*

	Composite psychiatric outcomes		Anxiety		Bipolar disorder		Depression		Sleep disorder	
	**HR (95% CI)**	***P*-value**	**HR (95% CI)**	***P*-value**	**HR (95% CI)**	***P*-value**	**HR (95% CI)**	***P*-value**	**HR (95% CI)**	***P*-value**
**Age**										
20–39	0.89 (0.65–1.26)	0.457	0.73 (0.44–1.19)	0.205	0.42 (0.10–1.79)	0.242	1.09 (0.74–1.62)	0.661	0.44 (0.16–1.23)	0.117
40–64	0.96 (0.82–1.13)	0.623	0.80 (0.62–1.03)	0.078	0.95 (0.45–2.01)	0.890	1.07 (0.85–1.35)	0.566	0.88 (0.64–1.22)	0.453
≥65	0.78 (0.67–0.90)	0.001	0.79 (0.63–0.98)	0.034	0.90 (0.45–1.82)	0.772	0.74 (0.59–0.93)	0.009	0.78 (0.58–1.03)	0.083
**Sex**										
Male	0.87 (0.73–1.04)	0.15	0.78 (0.59–1.02)	0.069	0.53 (0.19–1.48)	0.224	0.88 (0.67–1.16)	0.362	0.86 (0.61–1.20)	0.373
Female	0.84 (0.75–0.96)	0.008	0.78 (0.64–0.95)	0.011	0.99 (0.57–1.71)	0.971	0.91 (0.76–1.08)	0.281	0.74 (0.56–0.97)	0.027
**CCI**										
0	0.90 (0.69–1.19)	0.469	0.92 (0.62–1.38)	0.688	0.75 (0.23–2.47)	0.632	0.88 (0.58–1.32)	0.534	0.78 (0.44–1.39)	0.404
1	0.87 (0.71–1.07)	0.188	0.84 (0.61–1.15)	0.273	0.87 (0.30–2.52)	0.802	1.00 (0.74–1.36)	0.983	0.92 (0.62–1.36)	0.658
2	0.85 (0.69–1.04)	0.223	0.72 (0.52–1.00)	0.048	0.77 (0.30–1.97)	0.579	0.98 (0.73–1.32)	0.916	0.78 (0.51–1.20)	0.255
≥3	0.81 (0.68–0.96)	0.013	0.72 (0.56–0.93)	0.013	0.90 (0.41–2.01)	0.805	0.79 (0.62–1.01)	0.056	0.68 (0.48–0.98)	0.040
**Insurance type**										
Health insurance	0.86 (0.77–0.96)	0.007	0.81 (0.69–0.95)	0.010	0.98 (0.59–1.65)	0.944	0.89 (0.76–1.05)	0.163	0.77 (0.62–0.97)	0.024
Medical aid	0.80 (0.57–1.12)	0.194	0.47 (0.24–0.94)	0.032	0.39 (0.09–1.65)	0.201	1.00 (0.66–1.52)	0.989	0.90 (0.46–1.75)	0.755
Veterans' medical benefits	NE	0.999	NE	0.999	NE	NE	NE	NE	NE	NE
**CVD**										
No	1.05 (0.89–1.25)	0.551	0.88 (0.67–1.16)	0.375	1.11 (0.54–2.27)	0.780	1.20 (0.94–1.54)	0.146	0.95 (0.66–1.36)	0.774
Yes	0.78 (0.68–0.88)	<0.001	0.74 (0.61–0.90)	0.003	0.68 (0.35–1.32)	0.259	0.79 (0.65–0.95)	0.012	0.73 (0.56–0.94)	0.015
**DM**										
No	0.85 (0.76–0.96)	0.011	0.81 (0.68–0.98)	0.029	0.73 (0.40–1.34)	0.314	0.92 (0.77–1.10)	0.359	0.79 (0.62–1.01)	0.061
Yes	0.85 (0.71–1.03)	0.092	0.70 (0.52–0.94)	0.019	1.10 (0.49–2.47)	0.821	0.86 (0.66–1.12)	0.271	0.76 (0.51–1.13)	0.180
**CKD**										
No	0.84 (0.76–0.94)	0.001	0.78 (0.66–0.91)	0.002	0.87 (0.54–1.41)	0.573	0.90 (0.77–1.05)	0.171	0.77 (0.62–0.95)	0.015
Yes	1.14 (0.62–2.09)	0.676	0.67 (0.24–1.91)	0.458	0.00 (NE)	0.999	0.82 (0.32–2.14)	0.691	1.28 (0.43–3.88)	0.657
**Neurological disorders**										
No	0.85 (0.76–0.94)	0.002	0.78 (0.67–0.92)	0.003	0.80 (0.47–1.38)	0.432	0.90 (0.77–1.05)	0.191	0.77 (0.62–0.96)	0.020
Yes	0.88 (0.61–1.26)	0.485	0.68 (0.34–1.36)	0.276	0.92 (0.32–2.68)	0.877	0.84 (0.52–1.38)	0.494	0.90 (0.40–2.01)	0.799
**Benzodiazepine use**										
No	0.84 (0.74–0.96)	0.011	0.74 (0.60–0.91)	0.004	1.02 (0.52–2.00)	0.957	0.95 (0.78–1.16)	0.606	0.86 (0.67–1.11)	0.242
Yes	0.89 (0.76–1.05)	0.171	0.88 (0.69–1.13)	0.317	0.74 (0.37–1.49)	0.398	0.88 (0.70–1.10)	0.264	0.68 (0.46–0.99)	0.043
**Antiepileptic use**										
No	0.85 (0.77–0.94)	0.002	0.78 (0.66–0.91)	0.002	0.88 (0.51–1.51)	0.640	0.89 (0.77–1.04)	0.150	0.78 (0.63–0.96)	0.020
Yes	0.88 (0.51–1.50)	0.637	0.86 (0.30–2.45)	0.771	0.71 (0.25–2.03)	0.523	1.00 (0.49–2.04)	0.993	0.91 (0.21–4.07)	0.906
**Antidepressant use**										
No	0.82 (0.72–0.92)	0.001	0.73 (0.61–0.88)	0.001	0.93 (0.53–1.65)	0.812	0.87 (0.72–1.05)	0.144	0.76 (0.60–0.96)	0.022
Yes	0.98 (0.81–1.19)	0.845	0.97 (0.71–1.32)	0.851	0.71 (0.28–1.79)	0.463	0.98 (0.77–1.25)	0.881	0.92 (0.57–1.48)	0.731
**Antipsychotics use**										
No	0.85 (0.77–0.95)	0.004	0.79 (0.67–0.93)	0.004	0.80 (0.41–1.55)	0.509	0.90 (0.77–1.06)	0.206	0.79 (0.64–0.99)	0.037
Yes	0.77 (0.55–1.06)	0.110	0.61 (0.32–1.14)	0.118	0.82 (0.40–1.66)	0.575	0.83 (0.54–1.27)	0.381	0.56 (0.22–1.40)	0.213
**Hypnotics use**										
No	0.84 (0.76–0.94)	0.002	0.76 (0.64–0.90)	0.001	0.80 (0.47–1.35)	0.406	0.89 (0.76–1.05)	0.157	0.75 (0.59–0.95)	0.018
Yes	0.87 (0.64–1.18)	0.369	0.97 (0.58–1.62)	0.894	1.04 (0.30–3.63)	0.945	0.91 (0.58–1.42)	0.664	0.87 (0.56–1.34)	0.515
**Corticosteroid use**										
No	0.86 (0.78–0.96)	0.006	0.78 (0.66–0.92)	0.003	0.85 (0.52–1.39)	0.512	0.91 (0.78–1.06)	0.239	0.80 (0.64–0.99)	0.044
Yes	0.75 (0.51–1.10)	0.142	0.74 (0.42–1.29)	0.293	0.65 (0.08–5.16)	0.682	0.76 (0.43–1.36)	0.359	0.58 (0.25–1.35)	0.210

CCI – Charlson comorbidity index, CKD – chronic kidney disease, CVD – cardiovascular disease, DM – diabetes mellitus, HR – hazard ratio, IR – incidence rate, NE – not estimable, PY – person-years

*P-values are based on Cox proportional hazards models adjusted for baseline covariates.

### Sensitivity analyses

Sensitivity analyses using both IPTW and 1:1 PSM demonstrated similar overall findings with the main analysis (Table S1 and S2 in the **Online Supplementary Document**). Specifically, in the IPTW analysis, codeine use was associated with reduced risk of anxiety disorder (aHR = 0.83; 95% CI = 0.71–0.97) and sleep disorder (aHR = 0.76; 95% CI = 0.62–0.94). In contrast, in 1:1 PSM analysis, codeine use was associated with a lower hazard of anxiety disorder (aHR = 0.82; 95% CI = 0.68–1.00) and depression (aHR = 0.81; 95% CI = 0.68–0.98) compared with tramadol. A statistically significant association was observed only for anxiety in the ≥7-day subgroup (HR = 0.80; 95% CI = 0.64–0.99) in sensitivity analyses by cumulative opioid duration (Table S3 in **Online Supplementary Document****)**. There were no statistically significant associations for composite psychiatric outcomes, bipolar disorder, depression, schizophrenia, or sleep disorders across any exposure-duration thresholds.

## DISCUSSION

We compared psychiatric comorbidities associated with codeine and tramadol use in patients with hip OA, a population highly susceptible to persistent and severe chronic pain. Overall, codeine use was associated with a lower hazard of composite psychiatric comorbidities compared with tramadol, particularly for anxiety and sleep-related outcomes. Subgroup analyses further showed that lower hazard estimates were among those with a higher comorbidity burden (CCI≥3) or CVD, and those not receiving psychotropic or CNS-acting medications. These findings suggest potential differences in observed neuropsychiatric safety profiles of weak opioids.

Our findings should be interpreted in the context of the established association between psychiatric disorders and opioid dependence. Psychiatric comorbidities, such as depression, bipolar disorder, and schizophrenia, are among the strongest predictors of opioid misuse and opioid use disorder [9]. While pre-existing psychiatric comorbidities increase vulnerability to opioid misuse, we could not confirm that opioid exposure directly causes new-onset psychiatric disorders. Rather, we found differences in psychiatric outcomes between codeine and tramadol users. We observed higher hazards among patients with multiple comorbidities (CCI≥3), CVD, or those not receiving concomitant psychotropic or CNS-acting agents. These subgroup findings should be interpreted cautiously, as they may reflect differences in baseline vulnerability, prescribing patterns, or healthcare utilisation rather than definitive biological effect modification. Although tramadol’s serotonergic and noradrenergic activity may provide a pharmacologic context for these observations, mechanistic inferences cannot be drawn from claims-based data [15]. Among medically complex patients, we found lower hazard estimates for composite psychiatric outcomes with codeine compared with tramadol. While this may relate to differences in pharmacologic profiles, residual confounding cannot be excluded [16,17]. Duloxetine, a serotonin-norepinephrine reuptake inhibitor widely used for pain and depression, has been associated with neuropsychiatric and cardiovascular effects [18–21]. Considering tramadol’s partial monoaminergic activity, differential safety patterns may be plausible, though this interpretation remains exploratory. Clinically, these findings underscore the importance of careful psychiatric assessment and medication review when initiating weak opioids, particularly in medically complex patients, and support individualised opioid selection in routine practice.

Through subgroup analyses, we found that the association between weak opioids and psychiatric outcomes varied according to concomitant use of centrally acting medications, including benzodiazepines, anticonvulsants, antidepressants, antipsychotics, and hypnotics. Tramadol use was associated with a higher hazard of anxiety disorder and composite psychiatric comorbidities compared to codeine among patients not receiving psychotropic medications. Conversely, patients concurrently treated with hypnotics, antipsychotics, or corticosteroids had a lower risk of sleep disorders when using codeine. These findings should be interpreted with caution because, although we excluded patients with documented psychiatric diagnoses, the use of psychotropic agents may indicate subclinical symptoms or underlying vulnerabilities. Furthermore, prescribing indications for these centrally acting agents were not available in the claims data, and some medications may have been used for pain-related or other non-psychiatric conditions. Moreover, we did not capture baseline pain severity and functional impairment, and differences in pain burden may have influenced both opioid selection and psychiatric outcomes. Importantly, most of these CNS-acting medications are not recommended for routine concomitant use with opioid analgesics due to heightened risk of adverse effects, including excessive sedation, respiratory depression, cognitive impairment, and even mortality, which may signal inappropriate or unsafe prescribing practice [22,23]. Notably, co-prescription of opioids and benzodiazepines has been consistently linked to increased risk of opioid related overdose, implying the need for cautious medication review and strict adherence to prescribing guidelines [24]. These observations imply the importance of careful medication review and psychiatric monitoring in patients receiving opioid therapy. Further research incorporating detailed clinical data are needed to clarify these associations and assess whether integrated medication review and psychiatric monitoring can reduce opioid-related psychiatric harms.

The current Opioid Risk Tool does not identify sleep or anxiety disorders as major predictors for opioid use disorder [9]. However, sleep disturbances are highly prevalent among individuals with opioid use disorder, often manifesting as poor sleep quality, insomnia, and disrupted sleep architecture [24]. Similarly, anxiety disorders are prevalent in patients with opioid misuse [25]. Consistent with these observations, we found higher hazard estimates for sleep and anxiety disorders among tramadol users, suggesting that these conditions may warrant closer clinical attention in patients receiving opioid therapy. Considering tramadol’s serotonergic and noradrenergic properties, which can exacerbate neuropsychiatric symptoms, careful monitoring of sleep and anxiety symptoms may be appropriate in clinical practice. Further research is needed to determine whether incorporating sleep and anxiety assessment into routine opioid prescribing strategies improves patient outcomes.

Sensitivity analyses using IPTW and 1:1 PSM yielded broadly similar overall patterns, though statistical significance varied across the methodologies. We found reduced hazards for anxiety across multiple models, whereas associations for depression and sleep disorders were method-dependent. Furthermore, the lack of a duration-response relationship warrants cautious interpretation. Duration-restricted analyses indicated a statistically significant association for anxiety only in the ≥7-day subgroup, with no consistent associations across longer exposure thresholds. Several factors may explain these observed inconsistencies. First, we treated opioid switching as a censoring event. Patients who switched medications may have done so due to inadequate pain control or emerging side effects, both of which are strongly correlated with psychiatric distress. By censoring these individuals, the remaining cohort may represent a subset with greater tolerance or a better initial response, potentially diluting the observed risk over extended periods. Furthermore, the number of patients meeting longer cumulative exposure thresholds decreased progressively, potentially reducing statistical power in the ≥14-, ≥30-, and ≥60-day subgroups. As a result, the absence of statistically significant associations at longer durations should not be interpreted as definitive evidence of no effect but may partly reflect limited precision and wider CIs in these restricted samples. Further research validating the temporal relationship between opioid exposure and psychiatric outcomes is warranted.

Our findings add to the growing body of evidence indicating that safety profiles differ across individual opioids. In our study, codeine was associated with lower hazards for certain psychiatric outcomes compared with tramadol, which is consistent with previous reports suggesting lower risks of all-cause mortality, cardiovascular events, and fractures with codeine in specific comparative contexts [26]. However, other studies have shown that codeine may carry higher risks of all-cause mortality and cardiovascular outcomes when compared with oxycodone [27]. In addition, national surveillance data have identified tramadol as a leading contributor to analgesic-related adverse events in Korea [28]. Opioid stewardship programmes have been shown to improve prescribing appropriateness and minimise high-risk co-prescribing practices [29]. These findings support strengthening opioid stewardship efforts by incorporating structured psychiatric risk assessment and careful patient-centred opioid selection into routine practice. Further research is warranted to validate these comparative safety signals across diverse populations and to clarify their implications for clinical decision-making.

This study has several limitations. First, because of the retrospective design, residual confounding may persist despite PSM and sensitivity analyses. Particularly, baseline pain severity and functional status were not available in the database, and unmeasured differences in pain burden may have influenced both opioid selection and psychiatric outcomes. Additionally, physician prescribing behaviour and treatment preferences may have contributed to confounding by indication. Second, although ICD-10 codes were aligned with Diagnostic and Statistical Manual of Mental Disorders, fifth edition criteria, misclassification is possible because diagnoses were identified from administrative records. Some conditions may have been underdiagnosed or incompletely recorded, thereby attenuating observed associations. In addition, psychotropic medications may have been prescribed for non-psychiatric indications, introducing potential indication bias. Third, information on medication adherence and non-prescription medication use was unavailable, potentially introducing exposure misclassification and bias. Although we examined multiple psychiatric outcomes, these analyses were predefined and exploratory, and we did not apply formal multiple-comparison adjustment; therefore, the findings should be interpreted cautiously. Furthermore, tramadol was more frequently prescribed than codeine, requiring a 1:4 PSM approach, and we did not present subgroup interaction results for schizophrenia due to extremely low event counts and institutional data privacy restrictions. However, findings were generally consistent across IPTW analyses and 1:1 PSM. Moreover, because HRs are non-collapsible, unmeasured residual confounding, even if balanced at baseline, can attenuate HRs over time, and the observed relative risks should be interpreted as population-level associations. Finally, limited information on socioeconomic factors, family history, and lifestyle behaviours may have contributed to residual confounding, although the nationwide single-payer system reduces variability in healthcare access. Unmeasured provider-level factors and variation in clinical practice patterns may also have influenced treatment selection and outcome detection. Additionally, unmeasured factors such as lifestyle behaviours, provider prescribing preference, or unrecorded clinical conditions could have influenced the observational associations. Further studies are warranted to validate these findings across broader patient populations and to elucidate the biological and behavioural mechanisms linking opioid exposure to psychiatric outcomes.

## CONCLUSIONS

Codeine use was associated with a lower hazard of anxiety and a modestly reduced hazard of sleep disorders compared with tramadol, particularly among patients with a high comorbidity burden, CVD, or without psychotropic or CNS-acting medications. These findings imply that psychiatric safety, particularly with respect to sleep and anxiety disorders, should be considered when selecting among weak opioids. However, given the observational design and potential residual confounding, these associations should be interpreted cautiously. Opioid prescribing should therefore be individualised, considering patient comorbidity burden and concomitant medications. Further research is warranted to clarify how psychiatric conditions interact with opioid use trajectories and to inform safer, evidence-based pain management strategies.

## Additional material


Online Supplementary Document

## References

[1] GBD 2021 Osteoarthritis Collaborators Global, regional, and national burden of osteoarthritis, 1990-2020 and projections to 2050: a systematic analysis for the Global Burden of Disease Study 2021. Lancet Rheumatol. 2023;5:e508–22. 10.1016/S2665-9913(23)00163-7 37675071 PMC10477960

[2] Hall M van der Esch M Hinman RS Peat G de Zwart A Quicke JG How does hip osteoarthritis differ from knee osteoarthritis? Osteoarthritis Cartilage. 2022;30:32–41. 10.1016/j.joca.2021.09.010 34600121

[3] Lam S Amies V Hip arthritis presenting as knee pain. BMJ Case Rep. 2015;2015:bcr2014208625. 10.1136/bcr-2014-208625 25697301 PMC4336883

[4] Gibbs AJ Gray B Wallis JA Taylor NF Kemp JL Hunter DJ Recommendations for the management of hip and knee osteoarthritis: A systematic review of clinical practice guidelines. Osteoarthritis Cartilage. 2023;31:1280–92. 10.1016/j.joca.2023.05.015 37394226

[5] Li W He H Yang Z Wu Z Xie D Comparative risk-benefit profiles of weak opioids in the treatment of osteoarthritis: a network meta-analysis of randomized controlled trials. Postgrad Med. 2022;134:784–94. 10.1080/00325481.2022.2080360 35611671

[6] Martins SS Fenton MC Keyes KM Blanco C Zhu H Storr CL Mood and anxiety disorders and their association with non-medical prescription opioid use and prescription opioid-use disorder: longitudinal evidence from the National Epidemiologic Study on Alcohol and Related Conditions. Psychol Med. 2012;42:1261–72. 10.1017/S0033291711002145 21999943 PMC3513363

[7] Scherrer JF Svrakic DM Freedland KE Chrusciel T Balasubramanian S Bucholz KK Prescription opioid analgesics increase the risk of depression. J Gen Intern Med. 2014;29:491–9. 10.1007/s11606-013-2648-1 24165926 PMC3930792

[8] Scherrer JF Salas J Copeland LA Stock EM Ahmedani BK Sullivan MD Prescription opioid duration, dose, and increased risk of depression in 3 large patient populations. Ann Fam Med. 2016;14:54–62. 10.1370/afm.1885 26755784 PMC4709156

[9] Webster LR Webster RM Predicting aberrant behaviors in opioid-treated patients: preliminary validation of the Opioid Risk Tool. Pain Med. 2005;6:432–42. 10.1111/j.1526-4637.2005.00072.x 16336480

[10] Kang SH Kim HA Choi I Park CM Jhang H Kim J Psychotropic drug use in Korean patients with osteoarthritis. J Korean Med Sci. 2025;40:e53. 10.3346/jkms.2025.40.e53 40165576 PMC11964901

[11] Basham CA Edrees H Huybrechts K Hwang CS Bateman BT Bykov K Tramadol use in U.S. adults with commerical health insurance, 2005-2021. Am J Prev Med. 2024;67:558–67. 10.1016/j.amepre.2024.06.009 38876295 PMC11416325

[12] Miotto K Cho AK Khalil MA Blanco K Sasaki JD Rawson R Trends in tramadol: Pharmacology, metabolism, and misuse. Anesth Analg. 2017;124:44–51. 10.1213/ANE.0000000000001683 27861439

[13] von Elm E Altman DG Egger M Pocock SJ Gøtzsche PC Vandenbroucke JP Strengthening the Reporting of Observational Studies in Epidemiology (STROBE) statement: guidelines for reporting observational studies. BMJ. 2007;335:806–8. 10.1136/bmj.39335.541782.AD 17947786 PMC2034723

[14] Kim HK Song SO Noh J Jeong IK Lee BW Data configuration and publication trends for the Korean national health insurance and health insurance review & assessment database. Diabetes Metab J. 2020;44:671–8. 10.4093/dmj.2020.0207 33115211 PMC7643590

[15] Friedel E, Heinz A. Genetic, epigenetic, and environmental factors in serotonin-associated disease condition. In: Müller CP, Cunningham KA, editors. Handbook of Behavioral Neuroscience. Amsterdam, Netherlands: Elsevier; 2020. p. 923–940.

[16] Wang H Cheng Z Xu Z Wang M Sun X Liu W Systemic inflammatory factors and neuropsychiatric disorders: A bidirectional mendelian randomization study. Brain Behav. 2025;15:e70478. 10.1002/brb3.70478 40200869 PMC11979492

[17] Cătălina GR Gheorman V Gheorman V Forțofoiu MC The Role of Neuroinflammation in the comorbidity of psychiatric disorders and internal diseases. Healthcare (Basel). 2025;13:837. 10.3390/healthcare13070837 40218134 PMC11988559

[18] Maund E Guski LS Gøtzsche PC Considering benefits and harms of duloxetine for treatment of stress urinary incontinence: a meta-analysis of clinical study reports. CMAJ. 2017;189:E194-203. 10.1503/cmaj.151104 28246265 PMC5289870

[19] Park K Kim S Ko YJ Park BJ Duloxetine and cardiovascular adverse events: A systematic review and meta-analysis. J Psychiatr Res. 2020;124:109–14. 10.1016/j.jpsychires.2020.02.022 32135389

[20] Moon S Choo E Choi YJ Shin S Comparative cardiovascular safety of duloxetine and gabapentin in patients with hip osteoarthritis. J Pain Res. 2025;18:6437. 10.2147/JPR.S557621 41368597 PMC12682922

[21] Raj SR Stein CM Saavedra PJ Roden DM Cardiovascular effects of noncardiovascular drugs. Circulation. 2009;120:1123–32. 10.1161/CIRCULATIONAHA.107.728576 19770411 PMC2773827

[22] Hernandez I He M Brooks MM Zhang Y Exposure-response association between concurrent opioid and benzodiazepine use and risk of opioid-related overdose in Medicare Part D beneficiaries. JAMA Netw Open. 2018;1:e180919. 10.1001/jamanetworkopen.2018.0919 30646080 PMC6324417

[23] Siddiqui TG Cheng S Gossop M Kristoffersen ES Grambaite R Lundqvist C Association between prescribed central nervous system depressant drugs, comorbidity and cognition among hospitalised older patients: a cross-sectional study. BMJ Open. 2020;10:e038432. 10.1136/bmjopen-2020-038432 32718926 PMC7389767

[24] Ellis JD Mayo JL Gamaldo CE Finan PH Huhn AS Worsening sleep quality across the lifespan and persistent sleep disturbances in persons with opioid use disorder. J Clin Sleep Med. 2022;18:587–95. 10.5664/jcsm.9676 34569924 PMC8805005

[25] Rogers AH Zvolensky MJ Ditre JW Buckner JD Asmundson GJG Association of opioid misuse with anxiety and depression: A systematic review of the literature. Clin Psychol Rev. 2021;84:101978. 10.1016/j.cpr.2021.101978 33515811

[26] Xie J Strauss VY Martinez-Laguna D Carbonell-Abella C Diez-Perez A Nogues X Association of tramadol vs codeine prescription dispensation with mortality and other adverse clinical outcomes. JAMA. 2021;326:1504–15. 10.1001/jama.2021.15255 34665205 PMC8527363

[27] Lee J Kim Y Choo E Shin S Choi YJ Psychiatric, cardiovascular and skeletal risks of codeine versus oxycodone in hip osteoarthritis: A population-based cohort study. Drug Des Devel Ther. 2026;20:572083. 10.2147/DDDT.S572083 41710587 PMC12912106

[28] Choi YJ Kim MH Chung EK Lee JK Yoon J Yug JS Prevalence and seriousness of analgesic-induced adverse events in Korea: A 10-year nationwide surveillance. J Patient Saf. 2020;16:e215–24. 10.1097/PTS.0000000000000742 32604192

[29] Shoemaker-Hunt SJ Wyant BE The effect of opioid stewardship interventions on key outcomes: A systematic review. J Patient Saf. 2020;16:S36–S41. 10.1097/PTS.0000000000000710 32809999 PMC7447172

