# Supplementary material for: Comparative risk of psychiatric comorbidities associated with codeine and tramadol in patients with hip osteoarthritis: a nationwide population-based cohort study
Source: J Glob Health. 2026 Apr 24;16:04121. doi: 10.7189/jogh.16.04121 (PMC13105788; doi:10.7189/jogh.16.04121)
Supplement: Online Supplementary Document [file jogh-16-04121-s001.pdf]

Supplement to: Kim Y, Choo E, Shin S, Choi YJ. Comparative risk of psychiatric comorbidities associated with codeine and tramadol in patients with hip osteoarthritis: a nationwide population-based cohort study. J Glob Health. 2026;16:04121.

Table S1. Incidence rates and hazard ratios of psychiatric disorders and all-cause mortality in codeine versus tramadol users: inverse probability of treatment weighting (IPTW) analysis

Event counts are IPTW-weighted and may appear as fractional values

Outcome	Incidence Rate						aHR	
	Codeine			Tramadol			IPTW-weighted Cox models	
	Events	Total PY	IR/ 1000PY	Events	Total PY	IR/ 1000PY	aHR* (95% CI)	*P*-value
**Composite Psychiatric Outcome All** *	385	10,602	36.3	15,187	432,289	35.1	0.87 (0.78-0.96)	0.005
**Composite Psychiatric Outcome 1**	372	10,622	35	14,728	433,869	33.9	0.87 (0.78-0.96)	0.007
**Anxiety**	165	11,087	14.9	7,202	459,440	15.7	0.83 (0.71-0.97)	0.021
**Depression**	170	11,098	15.3	6,588	461,296	14.3	0.89 (0.76-1.04)	0.137
**Sleep Disorder**	90.2	11,271	8.01	4,305	469,189	9.18	0.76 (0.62-0.94)	0.012
**Composite Psychiatric Outcome 2**	16.4	11,434	1.43	918	479,869	1.91	0.63 (0.38-1.02)	0.060
**Bipolar disorder**	14.2	11,440	1.24	752	480,447	1.57	0.67 (0.40-1.14)	0.137
**Schizophrenia**	2.93	11,459	0.26	207	482,052	0.43	0.49 (0.15-1.54)	0.219

* Adjusted for age, sex, index year, insurance type, Charlson Comorbidity Index, comorbidities (hypertension, dyslipidemia, arrhythmia, heart failure, myocardial infarction, cardiovascular disease, diabetes mellitus, stroke, chronic kidney disease, chronic obstructive pulmonary disease, asthma, dyspnea, other respiratory diseases, pulmonary edema, musculoskeletal diseases, rheumatic diseases, osteoporosis, Parkinson's disease, Alzheimer's disease, epilepsy, other neurological disorders, peripheral vascular disease, sleep disorders, hyperthyroidism, hypothyroidism, liver disease, peptic ulcer disease, gastroesophageal reflux disease, inflammatory bowel disease, and other gastrointestinal diseases), history of medication abuse and fracture, and concomitant medications (benzodiazepines, anticonvulsants, antidepressants, antipsychotics, hypnotics, psychotropic drugs, non-opioid analgesics, non-steroidal anti-inflammatory drugs [NSAIDs], AAP, and corticosteroids).

Table S2. Incidence rates and hazard ratios of psychiatric disorders and all-cause mortality in codeine versus tramadol users: 1:1 propensity score–matched sensitivity analysis

Outcome	Incidence Rate						HR					
	Codeine			Tramadol			Crude		Adjusted*		Adjusted†	
	Events	Total PY	IR/ 1000PY	Events	Total PY	IR/ 1000PY	HR (95% CI)	*P*-value	HR (95%CI)	*P*-value	HR (95% CI)	*P*-value
**Composite Psychiatric Outcome All**	428	10,720	39.9	701	18,090	38.8	0.84 (0.74–0.95)	0.005	0.87 (0.77-0.98)	0.025	0.83 (0.73-0.94)	0.004
**Composite Psychiatric Outcome1**	412	10,747	38.3	684	18,142	37.7	0.83 (0.73–0.94)	0.003	0.87 (0.76-0.98)	0.024	0.83 (0.73-0.94)	0.004
**Anxiety**	177	11,265	15.7	331	19,380	17.1	0.78 (0.65–0.94)	0.009	0.82 (0.68-0.99)	0.041	0.82 (0.68-1.00)	0.049
**Depression**	202	11,237	18.0	331	19,344	17.1	0.85 (0.71–1.01)	0.070	0.90 (0.76-1.08)	0.269	0.81 (0.68-0.98)	0.029
**Sleep Disorder**	99	11,451	8.65	188	19,858	9.47	0.77 (0.60–0.98)	0.035	0.79 (0.62-1.01)	0.065	0.80 (0.62-1.03)	0.082
**Composite Psychiatric Outcome 2**	23	11,617	1.98	39	20,334	1.92	0.84 (0.50–1.42)	0.520	0.80 (0.46-1.38)	0.415	0.67 (0.37-1.22)	0.186
**Bipolar disorder**	19	11,626	1.63	32	20,361	1.57	0.87 (0.49–1.55)	0.637	0.83 (0.46-1.52)	0.554	0.67 (0.34-1.30)	0.233
**Schizophrenia**	5	11,650	0.43	11	20,402	0.539	0.62 (0.21–1.80)	0.378	0.44 (0.14-1.43)	0.173	0.41 (0.09-1.98)	0.269

* Adjusted for age, sex, Charlson Comorbidity Index, index year, insurance type, history of sleep disorder, and concomitant medication use (benzodiazepines,

anticonvulsants, antidepressants, antipsychotics, hypnotics, AAP, NSAIDs, and corticosteroids).

† Adjusted for age, sex, index year, insurance type, Charlson Comorbidity Index, comorbidities (hypertension, dyslipidemia, arrhythmia, heart failure, myocardial infarction, cardiovascular disease, diabetes mellitus, stroke, chronic kidney disease, chronic obstructive pulmonary disease, asthma, dyspnea, other respiratory diseases, pulmonary edema, musculoskeletal diseases, rheumatic diseases, osteoporosis, Parkinson's disease, Alzheimer's disease, epilepsy, neurological disorders, peripheral vascular disease, hyperthyroidism, hypothyroidism, liver disease, peptic ulcer disease, gastroesophageal reflux disease, inflammatory bowel disease, and gastrointestinal diseases), history of medication abuse and fracture, history of sleep disorder, and concomitant medication use (benzodiazepines, anticonvulsants, antidepressants, antipsychotics, hypnotics, psychotropic drugs, non-opioid analgesics, NSAIDs, AAP, and corticosteroids).

Table S3. Duration-restricted sensitivity analysis

Outcome	HR							
	≥7 Days		≥14 Days		≥30 Days		≥60 Days	
	HR (95% CI)	*P*-value	HR (95%CI)	*P*-value	HR (95% CI)	*P*-value	HR (95% CI)	*P*-value
**Composite Psychiatric Outcome**	0.92 (0.80–1.05)	0.202	0.84 (0.70–1.02)	0.078	0.93 (0.65–1.32)	0.679	0.75 (0.40–1.40)	0.363
**Anxiety**	0.80 (0.64–0.99)	0.039	0.77 (0.56–1.04)	0.085	0.68 (0.38–1.24)	0.211	0.66 (0.23–1.85)	0.430
**Bipolar**	0.96 (0.55–1.70)	0.896	1.29 (0.65–2.56)	0.470	2.51 (0.87–7.29)	0.090	0.00 (0.00–Inf)	0.999
**Depression**	1.01 (0.84–1.22)	0.893	0.87 (0.66–1.15)	0.338	0.93 (0.55–1.59)	0.802	0.71 (0.25–2.02)	0.526
**Schizophrenia**	0.66 (0.20–2.20)	0.495	1.11 (0.32–3.86)	0.871	2.18 (0.21–22.25)	0.510	0.00 (0.00–Inf)	0.999
**Sleep disorder**	0.80 (0.61–1.05)	0.101	0.75 (0.51–1.10)	0.139	0.88 (0.44–1.78)	0.726	1.31 (0.54–3.17)	0.555
